# A novel socially assistive robotic platform for cognitive-motor exercises for individuals with Parkinson's Disease: a participatory-design study from conception to feasibility testing with end users

**DOI:** 10.3389/frobt.2023.1267458

**Published:** 2023-10-06

**Authors:** Dor Raz, Shirel Barkan-Slater, Ilanit Baum-Cohen, Gal Vissel, Yeela Lahav-Raz, Amir Shapiro, Shelly Levy-Tzedek

**Affiliations:** ^1^ Department of Mechanical Engineering, Ben-Gurion University of the Negev, Beer-Sheva, Israel; ^2^ Department of Cognitive and Brain Sciences, Ben-Gurion University of the Negev, Beer-Sheva, Israel; ^3^ Tzeadim Neurorehabilitation Center and Parkinson’s and Movement Disorders Rehabilitation Unit, Sheba Medical Center, Beer-Sheva, Israel; ^4^ Department of Sociology and Anthropology, Ben-Gurion University of the Negev, Beer-Sheva, Israel; ^5^ Department of Physical Therapy, Recanati School for Community Health Professions, Ben-Gurion University of the Negev, Beer-Sheva, Israel; ^6^ Zelman Center for Neuroscience, Ben-Gurion University of the Negev, Beer-Sheva, Israel; ^7^ Freiburg Institute for Advanced Studies (FRIAS), University of Freiburg, Freiburg, Germany

**Keywords:** socially assistive robots, cognitive training, motor training, Parkinson’s disease, participatory design, feasibility testing, rehabilitation

## Abstract

The potential of socially assistive robots (SAR) to assist in rehabilitation has been demonstrated in contexts such as stroke and cardiac rehabilitation. Our objective was to design and test a platform that addresses specific cognitive-motor training needs of individuals with Parkinson’s disease (IwPD). We used the participatory design approach, and collected input from a total of 62 stakeholders (IwPD, their family members and clinicians) in interviews, brainstorming sessions and in-lab feasibility testing of the resulting prototypes. The platform we developed includes two custom-made mobile desktop robots, which engage users in concurrent cognitive and motor tasks. IwPD (n = 16) reported high levels of enjoyment when using the platform (median = 5/5) and willingness to use the platform in the long term (median = 4.5/5). We report the specifics of the hardware and software design as well as the detailed input from the stakeholders.

## 1 Introduction

Parkinson’s disease (PD) is a progressive neurodegenerative disorder caused by the degeneration of neurons in the substantia nigra, leading to a dopamine shortage (Lee et al., 2021) This results in a multisystem disorder affecting both motor and non-motor functions, including gait disturbances, dyskinesias, rigidity, sleep impairment, and cognitive decline (Sveinbjornsdottir, 2016). The resulting impairments often develop to be highly debilitating, preventing participation in many activities of daily living (Lingo VanGilder et al., 2021). PD is the second most prevalent neurodegenerative condition after Alzheimer’s disease, and its incidence is expected to increase to 8–9 million individuals in Western Europe by 2030 due to the aging of the world population aging (Dorsey et al., 2007; Driver et al., 2009). The median onset age of PD is 60–69 years old (Pagano et al., 2016) and approximately 10% of people with PD have young-onset PD (onset of symptoms is between 21 and 40 years of age) (Biddiscombe et al., 2020; Post et al., 2020).

Treatment methods for PD include the administration of drugs, such as levodopa (Pereira et al., 2019), and invasive surgery such as the implantation of deep brain stimulators (DBS) (Sveinbjornsdottir, 2016). However, the effect of both treatments decreases over time (Huot et al., 2013; Rizzone et al., 2014; Sveinbjornsdottir, 2016), and in the recent decades several complementary approaches have been studied, including physical exercise, music therapy and the “Train Big” approach and training of cognitive abilities such as the executive functions (Kalbe et al., 2020), as detailed below. Physical exercise in its various forms (including dancing and Tai Chi) has been recognized as complementary to traditional treatments in alleviating some of the symptoms of PD (Kurlan et al., 2015; Westheimer et al., 2015; Mak and Wong-Yu, 2019). Music has been suggested as a useful tool in accompanying rehabilitation exercises and has been shown to improve balance and gait stability as well to minimize anxiety and enhance wellbeing of individuals with PD (IwPD) (Pacchetti et al., 2000). Music can evoke both motor and emotional responses by simultaneously engaging multiple sensory pathways (Pacchetti et al., 2000). Music-based therapy for people with Parkinson’s disease is effective because it combines cognitive movement strategies, cueing techniques, balance exercises and physical activity while focusing on pleasure (de Dreu et al., 2012). Music Therapy (MT) has demonstrated the effectiveness of this approach on various aspects of affective, motor, and behavioral capabilities (Pacchetti et al., 2000). Cognitive training has been successful in improving the cognitive abilities of IwPD through, for example, computerized games (Petrelli et al., 2014) and online training (Petrelli et al., 2014; Fellman et al., 2020; Van De Weijer et al., 2020).

There is evidence that combining some of these approaches is also helpful. For example, combining movement (specifically, dance) training with music has been shown to improve cognitive skills and delaying the decline of executive functions and memory in IwPD (Pereira et al., 2019). Also, cognitive training before or concurrently with motor training has been suggested to optimize treatment outcomes for IwPD (Lingo VanGilder et al., 2021).

As the success of motor training relies on the adherence of IwPD to long-term exercise programs (Schootemeijer et al., 2020), effective interventions should motivate them to adhere to the training program. One approach could be to use gamified technologies, as has been implemented, for example, in cognitive training for healthy individuals (Cohavi and Levy-Tzedek, 2022).

Gamified exercise sets have been suggested as a means to encourage physical activity as they provide a pleasurable way of doing exercises (Adlakha et al., 2020). They have been shown to be effective as a complement to the traditional treatment of neurological disorders such as PD (Yuan et al., 2020). This has been the case in various contexts: gamification of rehabilitation treatments has been found to enhance the involvement and motivation of individuals after stroke to perform rehabilitation exercises (Zuki et al., 2022), to provide older adults with an opportunity to socialize with others while doing their exercises (Flores and Mataric, 2013), and to increase adherence in children with growth-hormone deficiency (Radovick et al., 2018). In the context of PD, several studies investigated the effects of gamified physical activity on parameters such as gait and cognition. For example, Pompeu et al. (2015) found an improvement of balance and gait performance with the use of Kinect games; Lopez et al. (2019) explored the acceptance of smart TV applications that successfully improved cognitive abilities of IwPD and individuals who suffer from different types of dementia; Cornejo Thum et al. (2021) implemented tele-rehabilitation with a treadmill that included virtual reality for patients with PD in their homes, and found that the system improved mobility and compliance with the training of the mentioned patients; Yuan et al. (2020) found that training with Virtual reality (VR) is effective in enhancing confidence in preventing falls for IwPD, as it improved posture and balance.

An underexplored technological tool for exercise for IwPD is the socially assistive robot (SAR). SARs have shown benefits in stroke (Feingold-Polak et al., 2021), cardiac rehabilitation (Casas et al., 2021), and acquired brain injury (Corallo et al., 2022), *inter alia*. It has yet to be determined whether they are able to increase engagement in exercise for IwPD. To the best of our knowledge, a single experiment to date has employed a SAR for IwPD, and was aimed at helping sort medications for IwPD (Wilson et al., 2020).

To investigate the perceptions of key stakeholders regarding the potential benefits and applications of robotic technology in aiding IwPD, we conducted focus groups involving different stakeholder groups. These groups included clinicians (Bar-On et al., 2023), as well as IwPD and their family members (Kaplan et al., 2023). These studies explored the needs, attitudes, concerns, and ideas related to technological interventions, specifically SARs, to support the PD population. These studies served as a basis for the current one.

Our goal in the present study was to develop and test the feasibility of using a SAR platform we developed for cognitive-motor training by IwPD. Ultimately, we aim to improve symptom management of those living with the disorder. As a first step towards this goal, we built a prototype of a robotic exercise platform, and collected input from IwPD and their family members on its strengths and weaknesses. The development of the platform was informed by: 1) the focus groups conducted with IwPD, their family members and their clinicians Kaplan et al., 2023; Bar-On et al., 2023), 2) the Training Big approach (Farley and Koshland, 2005), whose primary goal is to counteract the motor impairments experienced in Parkinson’s disease, such as slowness of movement and reduced range of motion; By stressing larger, exaggerated movements, the approach aims to enhance mobility, balance, and overall motor function (Farley and Koshland, 2005), and 3) the input we collected from stakeholders as the study unfolded through interviews, brainstorming sessions and questionnaires. To the best of our knowledge, this study is the pioneering effort to create SARs specifically for cognitive-motor training by individuals with Parkinson’s disease, using insights gathered from these individuals, their family members, and their healthcare professionals.

The overarching working hypothesis is that the training on the gamified cognitive-motor tasks will improve the patient’s clinical symptoms, with the robotic system serving as both the platform on which to practice, and as a motivating element. This will not be measured in the pilot experiments. The goal of the pilot experiments is to test the feasibility of using the device with IwPD, and to improve its usability prior to the larger-scale experiment to test our hypothesis.

## 2 Materials and methods

We built and tested the robotic platform using the participatory design approach. We designed it to help users perform motor-cognitive exercises, with the use of mobile desktop robots, light cues and music. By responding to the cues that the system provides, the user would ultimately practice working memory, inhibition, and sustained attention. In the motor domain, the system allows increasing range of movement and practicing movement precision.

In this section, we first provide the details of the physical setup, and then detail the participatory design process, including two phases of a feasibility test with IwPD. In the Supplementary Materials (Sections 1,2), we provide an overview of the hardware and software that we developed for the robots, including their subcomponents, algorithms for providing visual and verbal cues, moving and localizing in space, and logging clicks (user presses on the robot).

### 2.1 Physical platform

**FIGURE 1:**
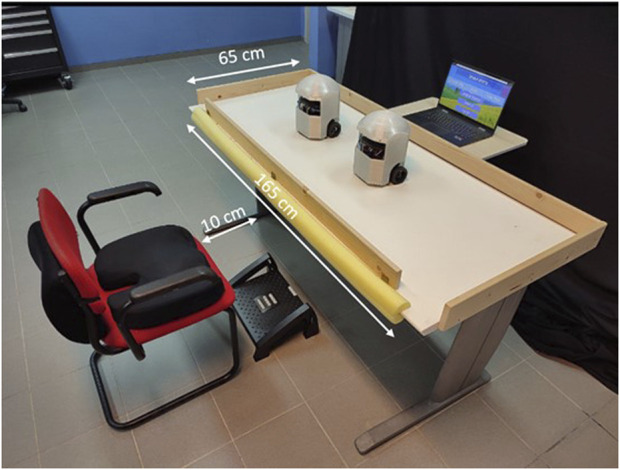
The physical setup of the cognitive-motor exercise games.

**FIGURE 2:**
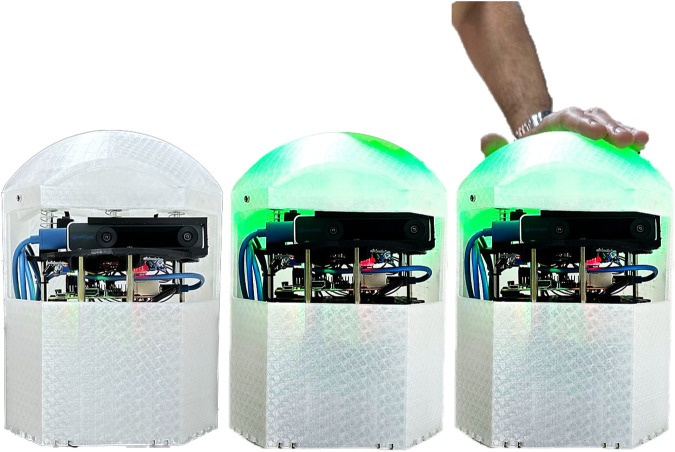
The AutoClicker mobile robot. The robot provides color prompts to the users and provides real-time visual and auditory feedback; It moves during some of the motor-cognitive exercises, such that users operate within a dynamically changing workspace. *Left*: the robot in its resting state (not pressed, no light cues); *Middle*: the robot lit up (green); *Right*: the robot is pressed down by the user.

The physical system setup can be seen in Figure 1. It includes a table, a comfortable sitting chair, a leg rest, and a small table for the speakers and the laptop. The game logic is implemented on the laptop, which communicates with the robots via Wi-Fi. The robots are equipped with microswitches to allow for pressing on them from the top (like a big push button). Linear motion shafts are connected to robot heads and used in conjunction with linear bushings and springs to facilitate accurate and linear clicking motion with minimal friction. Data from the camera, LED-matrix and microswitch are sent to the computer over Wifi. The prototype of the robotic platform, AutoClicker (AC), can be seen in Figure 2.

### 2.2 Game logic

**FIGURE 3:**
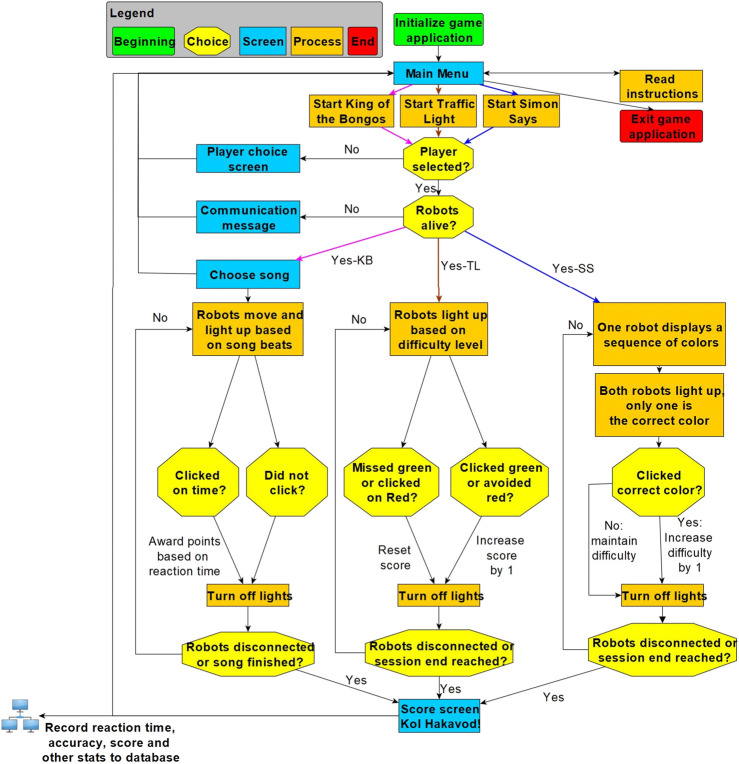
Flow diagram of the exercise sessions.

Figure 3 illustrates, using a flow chart, how the game application works. The game logic describes the steps and conditions by which an exercise session proceeds from beginning to end. The user starts at a “main menu” screen and may choose one of three practice session types or view instructive tutorials for them. Before choosing a session, a user’s name must be entered, or selected from a list of previous users to load the player’s progress and customized parameters as well as to update the correct player data in a custom-built database once the session is complete. Once a test has been automatically run to ensure that the robots are communicating with the PC application, the chosen session begins. Each session loops through a sequence of robot moves and light-ups followed by player clicks. Each session type has a unique stop criterion after which the game data are saved in a local database, and the user is returned to the main screen.

### 2.3 Experimental process: participatory design

The platform we present here is based on the results of focus groups held with IwPD and their family members (Kaplan et al., 2023) and their clinicians (Bar-On et al., 2023). One of the needs that transpired in those focus group discussions is for a robot to assist with cognitive and motor training.

**FIGURE 4:**
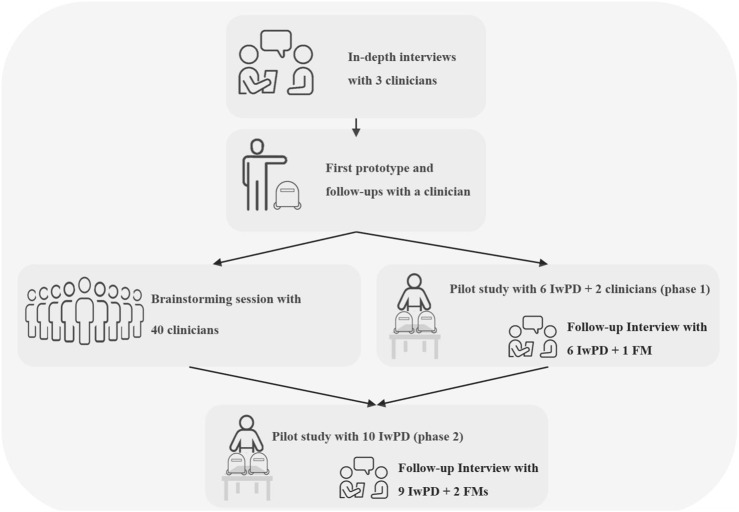
Participatory design workflow. Prototype 1.0 was used in Part 2. Prototype 2.0 was used in Part 3, during the pilot in Part 4 Phase 1 and also discussed in the Part 5 Phase 1 interviews. Prototype 3.0 was used in Part 4 phase 2 and discussed in the Part 5 Phase 2 interviews.

In the process of developing and testing the platform we subsequently built, we collected and implemented input from various stakeholders, including IwPD, their family members and their clinicians. This process is depicted in Figure 4.

We first held in-depth interviews with three clinicians. One of the clinicians was then provided with a preliminary prototype of the system, followed by a working prototype (prototype 1.0 described below). Afterwards, a brainstorming session with 40 clinicians was conducted in parallel to phase one of a pilot feasibility study; The pilot included practice sessions of IwPD with the robotic platform prototype 2.0, as well as interviews of the IwPD and their family members. Input from the pilot study as well as from the brainstorming session was implemented into a revised version of the prototype (3.0), used in Phase 2 of the pilot feasibility study. The two-phase feasibility test was conducted with a total of 16 IwPD and three family members. As a part of both phases of the pilot feasibility study, we conducted 15 semi-structured in-depth interviews with IwPD as well as with three family members.

#### 2.3.1 Part 1—in-depth interviews with clinicians

In part 1 of the participatory-design process, we held in-depth interviews with three clinicians: 1) an occupational therapist (OT1, Ph.D. candidate) at the institute for movement disorders and Parkinson’s rehabilitation at a large rehabilitation hospital located in the center of the country, with 12 years of experience treating IwPD; 2) A physical therapist (PT1, Ph.D) at the same institute with 12 years of experience treating IwPD; and 3) A physical therapist (PT2) with 14 years of experience treating IwPD at their homes and in groups.

The interviews were semi-structured and accompanied by slides, and were conducted via Zoom by author DR, a male Master’s student in Mechanical Engineering. OT1 was not part of the research team at the time that the interviews were held, and later joined the team (author IB-C).

#### 2.3.2 Part 2—first prototype and follow-ups with a clinician

Following the in-depth interviews, we designed a mock-up simulation of the proposed platform based on the input we received. In this simulation, moving dots resembling robots lit up in sync with the beat of a background song, while moving on a plane. We presented it to one of the clinicians (OT1) to get more input on the system. Based on the clinician’s input, we created a working prototype (prototype 1.0) and presented it to OT1 to get more feedback. This prototype included a short demo of the King of the Bongos exercise described below, with a single moving robot instead of two.

#### 2.3.3 Part 3—brainstorming session with clinicians

Once we had a working version of the updated prototype (prototype 2.0), we took it to a rehabilitation center in the periphery of the country (the Adi Negev rehabilitation center), where a wide range of patients, including IwPD, are treated. In order to collect feedback on the motor-cognitive exercise platform, we held a 1-h brainstorming session with the clinicians working in the rehabilitation center. The clinical team (n = 40) consisted of physiatrists, physical therapists, speech and language therapists, occupational therapists, psychologists, and social workers. The clinicians provided useful suggestions for improving the platform after we demonstrated its functionality.

This session took place in parallel with the first phase of the feasibility study. As a result of combining clinicians’ suggestions with the feedback collected in phase 1 of the feasibility study, we increased the difficulty level of the exercises, created a host-like behavior for the robot, and improved the synchronization of the movements of the robots with the music in order to increase difficulty and variety.

The results section lists the feedback and suggestions received in parts 1–3.

#### 2.3.4 Part 4—pilot feasibility studies with individuals with PD

Based on the suggestions we received from the clinicians, we designed the robotic exercise platform described in section 2.1 above. This system was then tested by IwPD to get the users’ perspective on the platform.

In addition to IwPD, phase 1 of the feasibility tests also included clinicians, so that their feedback can also be used to improve the system’s design. The pilot included a Registered Nurse (RN) and an Occupational Therapist (OT) who both work at the Soroka University Medical Center (SUMC).

##### 2.3.4.1 Study procedures

A total of 16 PD patients and two clinicians used the platform in a laboratory setting, and provided feedback on any changes that should be made to the system design; they were recruited in two phases: in Phase 1 six IwPD and two clinicians provided feedback on the system; we made the necessary changes to the robotic system based on their feedback, and then collected feedback from another group of 10 patients in Phase 2.

At the beginning of the session, upon arrival to the laboratory, the cognitive assessment of IwPD was conducted using the Montreal Cognitive Assessment (MoCA) test. The evaluation was performed by author SBS, a female Master’s student in the Department of Brain Sciences and Cognition, who holds a degree in psychology and has successfully completed the necessary online training for administering the MoCA test. The MoCA was not used for screening, but rather to provide a more detailed description of the characteristics of the participants. Each session lasted between 50–60 min. Of these, 25 min were dedicated to debriefing and using the exercise set, and 25–35 min for conducting the MoCA and answering the end-of-session questionnaires.

The platform offered three exercise types, each addressing a different aspect in motor-cognitive training, namely, motor control of the arms, working memory, inhibition and sustained attention (exercise sets “King of the Bongos” (KB), “Traffic Light” (TL) and “Simon Says” (SimS), respectively).

**FIGURE 5:**
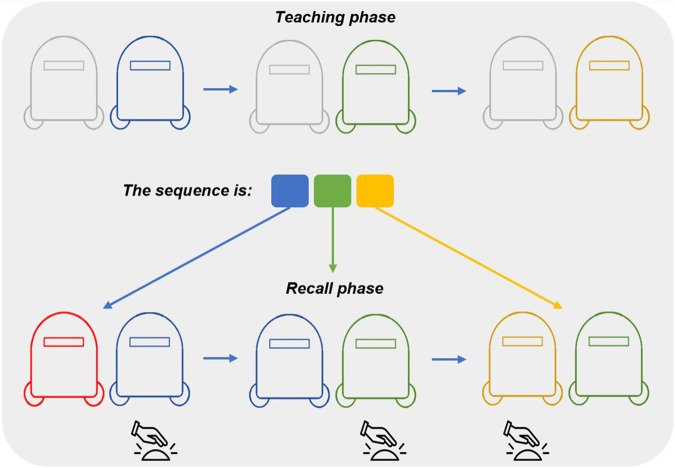
Simon Says exercise visualization. The first row shows the teaching phase, during which the “teacher robot” (the one on the right) lights up in a particular sequence (in this example, blue → green → yellow). The second row shows the recall phase, during which both robots light up in different colors, and the user should press either robot, once it is lit up with the correct color in the sequence (in this example, click the right robot for the blue and the green colors, and the left one for the yellow color).

Each participant completed one run with each of the three exercises:

I. King of the Bongos (3 min long)—the user chooses a song from a menu of songs. The song then starts playing and the robots light up in green in a rhythm which matches the beat of the song. The user is asked to press on the robots as they light up. The light fades out after 3 s or when the robot is pressed down. If the user presses the robots at the correct timing, they are awarded points based on their reaction time. Otherwise, no points are awarded.
II. Traffic Light (2 min long)—the robots light up in two different colors–red and green. Whenever a robot color is lit green, the user must press it, and whenever the color is red, the user must not press it. Pressing a green-lit robot, as well as not pressing a red-lit robot, awards the player a score of 1 point. The difficulty level of the exercise increases if the player is awarded multiple points in a row. The higher the difficulty level is, the shorter is the duration between light-ups, as well as the light-up durations. If the user fails to press a green-lit robot, or presses a red-lit robot, the score resets back to 0 and the difficulty level decreases by 1.
III. Simon Says (3 min long)—this sequence-learning exercise is composed of pairs of two stages–the teaching stage and the recall stage (Figure 5).
  a. In the teaching stage, one of the robots (the Teacher) lights up in a sequence of colors. The length of the sequence depends on the difficulty level: at the simplest level, the sequence is two colors-long, and at each additional level another color is added to the sequence (up to a maximum of 5 colors). Once the teaching stage is over, the recall stage begins.
  b. In the recall stage, each of the two robots lights up with a different color–one of which is the correct color in the pattern given by the Teacher. The patient must press the correct colors in the correct order to advance to the next level of difficulty. If they are successful, the level of difficulty will increase, and if not, it will remain the same.

After having trained using all mode types, the patient will be asked to choose the preferred mode to play for the final 3-min set.

Following the training sets, the participants were asked to fill a questionnaire about the rehabilitation platform–to rate their perceived level of engagement with the platform, its perceived benefits, areas for improvement, and their intention to use it in the long term.

In order to reduce potential bias in the users’ perception of the platform (e.g., users may be inclined to choose the most recent exercise set as their final exercise), participants were semi-randomly assigned to a training sequence (A-B-C, A-C-B, B-A-C, B-C-A, C-A-B, or C-B-A), with A denoting the King of the Bongos exercise, B denoting the Traffic Light exercise and C denoting the Simon Says exercise.

The experimental protocol was approved by the Human Subjects Research Committee of Ben-Gurion University, and all participants gave their written informed consent to participate in the study.

##### 2.3.4.2 Outcome measures

We asked participants to rate their perceived level of engagement with the platform, its perceived benefits, areas for improvement, and their intention to use it in the long term. We also measured reaction time, and precision on the task.

#### 2.3.5 Part 5: in-depth interviews with IwPD and family members

In parallel with the pilot feasibility studies, we conducted a total of 18 semi-structured in-depth interviews: 15 with IwPD who participated in the pilot study and three with family members of IwPD who participated in the pilot study. The aim was to capture the participants’ perceptions and experiences (Denzin et al., 2017) with the SARs and how it may assist their specific needs; we were also interested in their willingness to use it at home or in the clinic. The study was approved by the university’s ethics committee and the Helsinki committee of the Soroka Hospital.

The interviews were conducted shortly after participants took part in one of the pilot feasibility studies (ranging from immediately at the end of the session with the robotic platform to 2 weeks after it, based on the availability of the participants and the interviewer). The place of the interview was determined according to the interviewee’s preferences. Seven interviews took place in the laboratory immediately after the end of the experiment (of those, one was continued via zoom, and two were continued at the participants’ home), nine took place on Zoom, and two in the interviewees’ homes.

All interviews were conducted by author GV, a male bachelor’s student in Sociology and Anthropology, with the guidance of author YLR, an experienced qualitative researcher.

Data analysis followed the stages proposed by Strauss and Corbin. (1998). In order to identify themes and concepts, we hand-coded the interview transcripts using open and *in vivo* coding techniques. We grouped and regrouped the resulting themes using axial coding, until the key categories were identified. We separately analyzed each key category in order to gain a deeper understanding of the participants’ perceptions and experiences.

## 3 Results

### 3.1 Parts 1–3 iterative design process

Supplementary Table S1 in the Supplementary Materials summarizes the results from parts 1 (in-depth interviews with clinicians), 2 (prototype 1.0 and follow-ups with a clinician) and 3 (brainstorming session with clinicians). The feedback we received from the participants is categorized as follows: 1) User interface and experience (e.g., the need to simplify the information displayed on the screens so they are not distracting); 2) Customization and flexibility (e.g., the need to give a choice of multiple songs for added variability); 3) Design and aesthetics (e.g., suggestions on the physical design features of the robots and their gestures); 4) Music and audio feedback (e.g., add a “good job”/”oops” sound for immediate auditory feedback); 5) Exercise modes (e.g., the need to slow down the pace of the exercise); 6) Data visualization (e.g., an emphasis on the need for graphic visualization for clinicians); 7) Experimental design (e.g., ideas for adjustments to the inclusion and exclusion criteria); 8) Safety (e.g., ensuring the experimental setup does not pose a risk of falling); and 9) Ideas which we did not implement (e.g., specific suggestions on how to use the robot for speech-language therapy).

### 3.2 Part 4—pilot feasibility study with individuals with PD

In the following sections we detail the responses from participants in the two phases of the feasibility study, regarding the platform and its potential use in the short and long term. The baseline characteristics of the IwPD who took part in phases 1 and 2 of the feasibility study are listed in Table 1.

**TABLE 1:** Participant baseline data. Data are shown for the 16 IwPD who participated in Phases 1 and 2 of the feasibility testing. The participant code is composed of the Phase (S1/S2 to denote Phase 1/Phase 2, respectively) and the participant’s consecutive number within the phase.

Participant code	Age [years]	Gender	MOCA	Years since PD onset
S1P1	69	Man	27	5
S1P2	66	Man	30	7
S1P3	63	Woman	28	3
S1P4	48	Woman	27	1
S1P5	45	Woman	26	1
S1P6	68	Man	24	5
S2P1	40	Man	27	5
S2P2	75	Man	26	19
S2P3	68	Woman	27	0
S2P4	72	Man	23	11
S2P5	63	Woman	26	5
S2P6	68	Woman	29	6
S2P7	72	Man	26	2
S2P8	72	Man	26	3
S2P9	69	Man	30	8
S2P10	71	Man	24	6

#### 3.2.1 Phase 1—pilot study with 6 individuals with PD and 2 clinicians (RN + OT)

Eight overarching themes emerged from the users’ responses on using prototype 2.0:

##### 3.2.1.1 Physical setup (display size, comfort, etc.)

Two participants referred to the comfort of the chair and its distance from the exercise table (which suited one but not the other). Four noted that the setup is too big and cumbersome to be used in a private home.

##### 3.2.1.2 Robot physical design (clickability, human-like design features, etc.)

Two participants stated their preference for robots that have added design features, (e.g., with an added mustache to make it more amusing and appealing, or designed as pairs–such as a boy and a girl); they also suggested to completely hide the electronics behind the casing. One participant felt that the robot’s tall height was beneficial for training the upper body but is too big for a finalized industrial product.

##### 3.2.1.3 The tasks (difficulty, variability, engagement, etc.)

Two participants found the background music in the Simon Says exercise to be distracting, and one found the exercise to be stressful. Four thought the exercises were not sufficiently challenging, two of them said that they would use a system with more challenging levels at home. Two participants thought the robots should move more and at different speeds and patterns in the King of the Bongos exercise to increase engagement.

##### 3.2.1.4 Reasons for choosing final task (difficulty, potential improvements, weak spot, etc.)

Three participants who chose TL said the exercise was simple enough to be clear and engaging. One participant who described herself as competitive chose Simon Says to prove to herself that she can improve and win albeit the high difficulty. Two participants chose KB since the music and the spatial movements helped them engage and focus throughout the exercise.

##### 3.2.1.5 Engagement in the short term

Two participants expressed their liking to the music and the engaging spatial movement of the robots in the KB exercise, and five of the six participants marked it as their most liked exercise (Figure 10). One participant described the TL exercise as “positively tiring” for the hands, especially the dominant one. Another said the movement in the KB exercise improved the attention span and increased engagement in the game.

##### 3.2.1.6 Engagement in the long term

Four participants replied they would use the platform at home, one replied that it was not challenging enough and another that it was not exciting enough. Four participants explained that the system needed to be more engaging and variable to maintain motivation to use it over a prolonged period of time. Two of them suggested that more challenges or competition with other users would be necessary to maintain motivation over time. According to one participant, external motivation is necessary in order to begin practicing with the system at home. One participant indicated that she would be very interested in using the system over the long term.

##### 3.2.1.7 Social aspect of the platform

One participant expressed a desire for the robots to greet him and to instruct him on how to perform the different exercises (rather than have the research team do it). Two other participants also suggested adding a competitive mode, which can be played with other participants in person or through the web, as a way to increase motivation. A fourth participant stated that the mere act of coming to the lab and meeting its members made her feel like a part of a family and want to come again.

#### 3.2.2 Phase 2—pilot study with 10 individuals with PD

Eight overarching themes emerged from the users’ responses to the questionnaire regarding the use of the prototype 3.0:

##### 3.2.2.1 Physical setup

It was suggested by three participants that future prototypes should be smaller. One participant requested a larger screen for displaying the game score.

##### 3.2.2.2 Robot physical design

Three participants suggested that the robot’s clicking mechanism should be strengthened. Two thought the robots should be smaller and shorter, and another thought the robots should be closed off to hide the electronics behind the casing.

##### 3.2.2.3 The tasks

Participants felt that the SimS tutorial required better explanations and perhaps a brief demonstration. The SimS game was deemed to be too difficult by one participant. On the other hand, two others expressed a desire for all exercise sets to be more challenging. According to one participant, the robots moved too far apart and were difficult to track in the KB exercise set, whereas another thought they should move throughout all three exercises.

##### 3.2.2.4 Reasons for choosing final exercise set

Figure 11 shows the exercise sets that participants chose as their final exercise session in the two phases. Eight out of ten participants in Phase 2 selected the KB exercise. It was chosen by one participant because it amused him, by two participants because they enjoyed the music, and by another participant because it required a lot of movement as well as hand-eye coordination. Others felt it was intuitive and simple to play as a final exercise.

##### 3.2.2.5 Engagement in the short term

Three participants found the exercises to be engaging, mesmerizing, and full of action. Another participant commented that, despite the exercises not being challenging, he enjoyed participating in the experiment and getting to know the lab members. One participant thought the music helped alleviate the shaking in his hands, and another noted that the system assisted him in moving the hand more affected by PD as the session progressed. His granddaughter, who accompanied him to the experiment, said afterwards: “I have never seen my grandfather active like this, I was in tears.”

##### 3.2.2.6 Engagement in the long term

A total of eight participants expressed an interest in engaging with the platform in the long term. One participant conditioned his participation on a system with smaller robots, and another on a more challenging exercise. Conversely, a participant residing near the laboratory preferred using the platform on-site rather than at home. Two other participants noted that certain households do not have suitable tables or computers, and that future systems must be user-friendly in order to be successful. According to one participant, a greater musical variety is needed over the long term. A second suggestion was to add more difficulty levels to the exercises, and a third suggested awarding prizes for high scores to increase motivation to practice.

##### 3.2.2.7 Social aspect of the platform

Two participants suggested that the robots greet them in a friendly manner and recognize them. One participant indicated that he would prefer to train with a human, while another stated that he would prefer to practice with three robots rather than two.

##### 3.2.2.8 Suggestions and observations

Two participants noted that it would be more beneficial to practice standing up, rather than sitting down.

##### 3.2.2.9 Outcome measures

**FIGURE 6:**
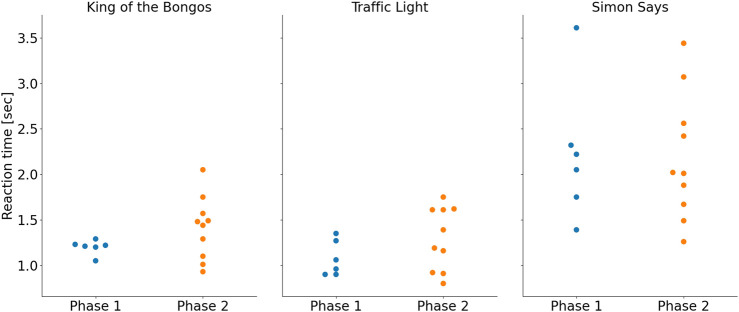
Average participant reaction times for each exercise. Results for Phase 1 are shown in blue (left) and for Phase 2 in orange (right).

**FIGURE 7:**
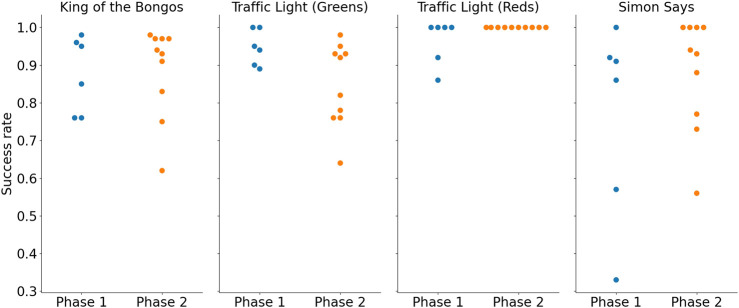
Average participant success rate for each exercise. Results for Phase 1 are shown in blue (left) and for Phase 2 in orange (right).

Figure 6; Figure 7 show the average participant reaction time and success rates, respectively, for all three exercise sets. It appears there was a trend of increase in reaction time from Phase 1 to Phase 2 in the KB and the TL exercise sets (KB: 1.2 ± 0.1 s vs. 1.4 ± 0.3 s; TL: 1.1 ± 0.2 s vs. 1.3 ± 0.3 s; SimS: 2.2 ± 0.7 s for both phases). Success rates were maintained in the KB exercise (87% ± 9% in Phase 1% vs. 88% ± 11% in Phase 2), and increased in the SimS exercise (76% ± 23% vs. 88% ± 14%). There were opposing trends within the TL exercise: the success rate for clicking the green lights when they turned on went down (94% ± 4% vs. 84% ± 10%), whereas the success rate for avoiding clicking the red lights when they turned on went up (96% ± 5% vs. 100%).

These trends were not statistically evaluated due to the small number of participants.

**FIGURE 8:**
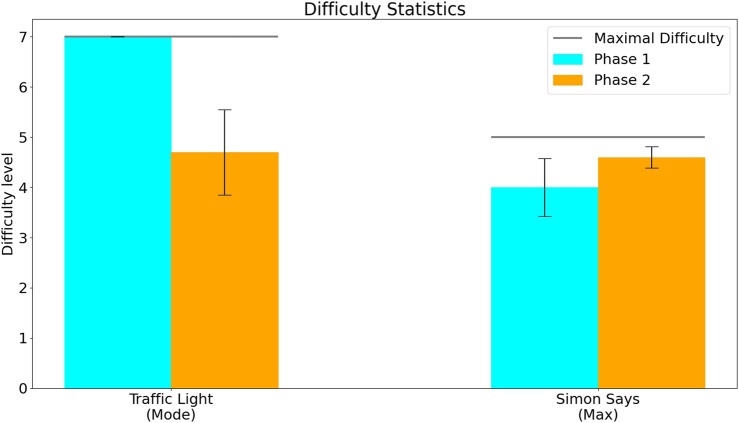
Difficulty level reached by participants in the two phases. The difficulty level in the Traffic Light exercise (left) shows the mode–the difficulty level at which the participants spent the most time (since the difficulty level of this exercise could both increase or decrease depending on user performance). The difficulty level in the Simon Says exercise (right) shows the maximal difficulty level that participants reached (since the difficulty level for this exercise only increased based on user performance, but did not decrease).

**FIGURE 9:**
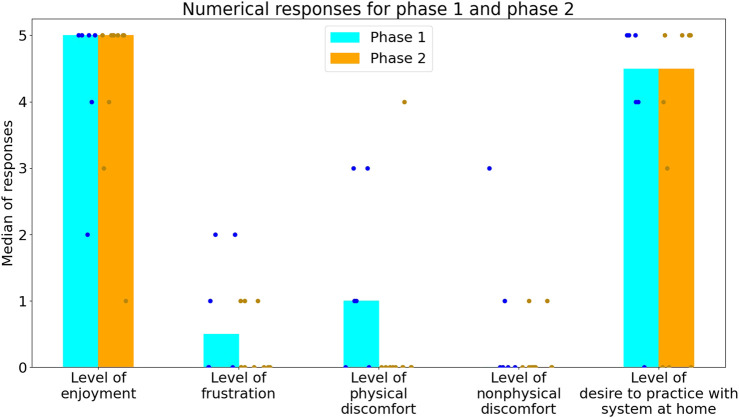
Participants’ rating of their experience, on a scale of 0–5. The bars denote the median response, and the dots represent the individual responses of the participants (blue for Phase 1 and orange for Phase 2).

**FIGURE 10:**
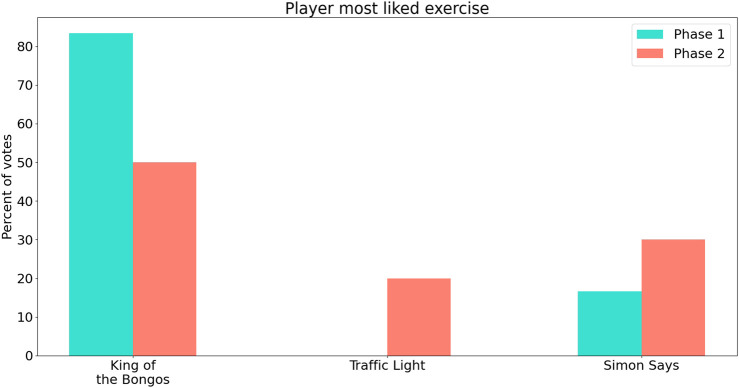
Participants’ most liked exercises in the two phases. Results for Phase 1 are shown in blue (left) and for Phase 2 in pink (right).

**FIGURE 11:**
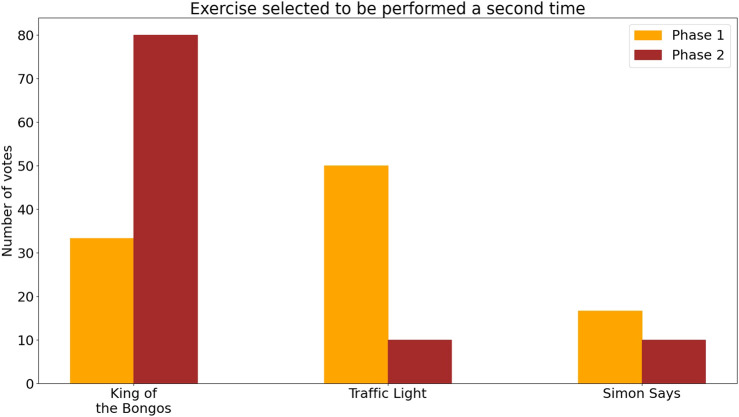
Participants’ choice of an exercise set for the final session in both phases. After training with all three exercise sets, participants were asked which exercise they would like to do as the fourth and final exercise session. Results for Phase 1 are shown in orange (left) and for Phase 2 in red (right).

Figure 8 shows the maximal difficulty level and mode difficulty level reached for the TL exercise as well as the maximal difficulty reached in the SimS exercise. Figure 9; Figure 10; Figure 11 show the participant responses to the questionnaires in both phases.

### 3.3 Part 5: in-depth interviews with IwPD and family members

Interviews revealed that a significant struggle faced by IwPDs is keeping social ties with other IwPDs and the rest of society, who do not necessarily understand what IwPDs cope with. Therefore, when designing a system to help with PD treatment, it is important to look at the social factor and consider how SARs may contribute to IwPD’s ability to create and maintain social connections. We identified five themes relevant to the social factor and how they impact IwPDs’ acceptance of the robot and their willingness to use the assistive robot in their home environment. These include the participant’s conciliation with their diagnosis, the participants’ illness stage, age, familial status and the robot’s potential influence on the caregiver’s routine.

#### 3.3.1 Participant’s conciliation with their diagnosis

Analysis revealed a connection between participants’ willingness to use the robot and their conciliation with their disease. Those who expressed hardships and who tended to hide their diagnosis (e.g., trying to hide their symptoms when in the presence of others) were keener to use the robots in their home environment. They were not keen to use the robots publicly and stated that they would not come to a rehabilitation center to use them.

• “When I walk with a group, I wrap my hand with a long scarf, that way no one sees if my hand is trembling … No one knows I have PD … The robots can be in my house and in a public center, but I would prefer to have it in my house”. (Olivia, age 68)
• “When I was diagnosed, I found it difficult to accept it, and I still find it difficult now … I think the robots will be most effective in the house” (Alex, age 72)

On the contrary, others, who expressed acceptance of their illness, were more willing to use robots, regardless of their location. They were more comfortable and even preferred practicing with other people rather than by themselves:

• “If the robot would cause four, eight, or even ten people to sit and play together, for me, it would be wow!”. (John, age 69)
• “It could be nice if you connect it to social media and play against others … a type of competitive game”. (Maria, age 48)
• “It should bring a few people; even hold a small competition and practice together.” (Robert, age 76)

#### 3.3.2 Participant’s illness stage

While participants’ conciliation with their diagnosis and their illness stage do not necessarily coincide, our data reveal a connection between participants’ illness stage and their willingness to use the robots further. Those who were diagnosed more recently and experienced initial symptoms felt that the robots were not relevant for them in dealing with their symptoms:

• “I am not sure that I am the best prototype user because I am almost fully and independently functional”. (Maria, age 48)
• “In my condition today, I do not feel that it is relevant for me”. (Charles, age 72).
• “As an IwPD today, I did not feel challenged [during practice with the robots]”. (James, age 47)

However, participants that indicated they suffer from severe symptoms, were keener to use the robots. They felt that the robots were more relevant to both their physical practice, cognitive abilities and their social activities:

• “I will benefit from this technology at home” (John, age 69).
“I think it is relevant … I will use the robot at least once a day at home” (Mark, age 71)

##### 3.3.2.1 Age

Our findings indicated that the willingness to use the robot was closely connected to age. Out of sixteen participants, thirteen of whom were at the age range 63–76, three were younger (in their forties). The younger ones repeatedly emphasized that the robots are not relevant to them, and they found them to be not challenging both in terms of cognitive level and in terms of motor abilities:

• “I am not sure I am the best prototype user since I am at an almost fully functional level. However, for people who struggle daily, I think it can be useful”. (Maria, age 48).

Those who were in a later stage of the disease were all above the average age of the participants, and as previously mentioned, they felt the robots were relevant to them. They stated they could benefit from the practice both in a clinic as a means to meet with others and at home as a treatment tool.

• “If I had space in my house, I would want to use the robots to practice … Although practicing with people is not in my character, I will try. I think the best way to practice with people, is through the internet” (Richard, age 69)
• “I will use it with other people, even in a competition with ten people”. (Robert, age 76)
• “I like being at home, so I would use it at home. My wife will make sure I will use it every day”. (Oliver, age 75)

It is possible that the difference along the age continuum in fact reflects a difference in disease stage, which we did not record in this study. While we did not collect information about the participants’ disease stage directly, there is a moderate correlation between the age and the years since PD onset in the participants of this study (R = 0.41, *p* = 0.11; see Table 1).

##### 3.3.2.2 Familial status

The younger the patients were and the more occupied they were with caring for young children, the less they were motivated to use the robots. For older patients, in contrast, practicing with the robot actually reminded them of their grandchildren, and they expressed a positive perception of using a robot:

• “It can be nice with the kids, not only IwPD … with grandchildren, it is a competition” (Emma, age 63).

Hence, they enunciate the potential social effect of using the robot, vis-à-vis the younger generation, by, for example, showing their grandchildren their relevance and connection to novel electronic devices.

##### 3.3.2.3 The robot’s potential influence on the caregiver’s routine

Although we conducted only three interviews with family members who serve as the participant’s main caregivers, our findings reveal the potential impact of the SARs on alleviating the load and burden they face on a daily basis. They highlighted their challenges of always having to be alert, be constantly present and assist with daily tasks. Thus, when thinking of the SARs as a treatment tool that participants can hopefully use by themselves, they stated it could provide them time for rest, both physically and mentally:

• “It gives you peace of mind that there is something else, which is not you, that can help him [William] progress”. (Lisa, spouse of William)

It is important to note that we held only three interviews, and so further research, which focuses on FM, is required in order to understand the benefits these robots can have on their routine.

#### 3.3.3 Practical aspects regarding the use of the platform

Interviews analyses revealed two sub-themes concerning the system’s usage at the participants’ houses and treatment centers. The first is the simplicity of the user interface, and the second is the speech factor.

##### 3.3.3.1 User interface

Beyond the robots’ design, an easy-to-use interface that lets one operate the system seems crucial to participants. when asked about their experience in the laboratory, participants responded that the system worked well. However, they also stated that it had to be more interactive:

• “The interaction should be simpler, interactive and friendlier such as greeting [the users] or creating social dialogue [with them]. If it remains only explanatory, I find it harder to stay in focus. (Emma, age 63)

Other older participants also stated they fear using technology and expressed concerns about the complexity of the system and about using it by themselves if the robot will be placed at home:

• “How will I be able to activate it by myself?” (William, age 68)

It exemplifies participants’ fear of having to use the system at their homes without assistance. Participants also explained that the system needs to articulate and demonstrate the assignment, thus making it easier to use in laboratories or treatment centers.

##### 3.3.3.2 The robots’ speaking abilities

Participants were more willing to accept robots who could speak with them. They expressed a desire to have a conversation with the robots during training, to be greeted and receive encouragement from them, and to have their questions answered by the robots. Participants referred to the ability to speak as a crucial factor in their sense of connection to the robot:

• “It would be nice to talk to him, and he can help me practice … my wife will find tasks for him in the house”. (William, age 68)
• “I would love it if I had a robot that talks to me and understands what I say” (John, age 69)

The robot’s speaking abilities were also highlighted as attractive by family members. For example, Lisa, the spouse of William, said: “[the robot] reminds him to drink or take his medicine. He should become a part of the IwPD’s daily routine.”

Hence, attributing human qualities to robots can help people connect with them and encourage them to practice more, thus, transcending the boundaries of the robot as a medical tool. Participants explained that the system has to be more interactive in order to make the user feel comfortable about using it:

• “When there is an interaction with the computer, simple explanations … it is easier and contributes more to the practice” (Emma, 63).
• “I would maybe give him [the robot] a human face, a smile. Something that will be nice … Maybe if the robot could talk, it would be more human”. (Nicole, Spouse of Oliver)

Moreover, the presence of the laboratory staff, and the extra explanation they provided, proved to be important to people’s ability to fully understand the instructions. Therefore, providing more interactive speech abilities and explanations seems crucial to make the system accessible to the users, especially if it is intended for use at home.

## 4 Discussion

In this study, we developed and tested a social robotic platform for cognitive-motor exercise using the participatory design approach with input from a total of 62 stakeholders (IwPD, their family members and clinicians). The iterative process we employed, along with a mixed-methods approach, enabled us to repeatedly improve the design of the platform, such that it better matches the needs of the users. The users in the two parts of the feasibility study reported high levels of enjoyment (median = 5) and of willingness to continue training with the platform (median = 4.5). While these numbers show the general trend, the youngest users in each phase (S1P5, aged 45; S2P1, aged 40) were found to be outliers in terms of their enjoyment using the system (using the inter-quartile range method) reporting level of enjoyment of 2 and 1 accordingly. S1P5 was also found to be an outlier with a reported level of 0 willingness to use the system at home.

In accordance with principles of effective rehabilitation (Maier et al., 2019), we implemented the following aspects in the training: repetitive and goal-oriented practice, variable difficulty, and rhythmic cueing. The exercises simultaneously trained cognitive and motor aspects within a gamified environment, and the difficulty levels of the exercises was automatically adjusted based on the user’s performance, and logged along with success rates.

In the cognitive domain, the system aimed to assist users in training their ability in the areas of inhibition, short-term memory, and sustained attention. IwPD often experience difficulties in effectively filtering distractors (McNab and Kleinberg, 2008; McNab et al., 2015), which is a critical component of their working memory capacity. In the motor domain, the platform required extended reaching movements of the shoulder, predominantly flexion, horizontal adduction, horizontal abduction, and protraction arm movement, specifically encouraging movements exceeding 90°. These are crucial for performing activities of daily living (ADL) such as combing one’s hair and washing one’s back (Triffitt, 1998).

According to the definitions put forth by Fasola and Mataric. (2013), ”a socially assistive robot (SAR) is a system that employs hands-off interaction strategies, including the use of speech, facial expressions, and communicative gestures, to provide assistance in accordance with a particular healthcare context”. Our robots’ utilization of speech (e.g., “Let’s start playing!”) and movement that invites the user to interact with them aligns with Fasola and Mataric’s characterization of SARs. Throughout the session, the robots are able to adapt the difficulty level based on patient performance, as well as mimic a social dancing gesture by moving in synchronization with songs. These illustrate their socially interactive nature.

Additionally, our robots’ functionality aligns with another core function of assistive robotics - providing assistance to users in diverse contexts, such as rehabilitation healthcare. Our robots’ ability to provide cognitive-motor exercise, while monitoring the user’s performance, providing feedback to the user, and displaying performance levels over time, to be used by patients and clinicians in the future, is in line with Feil-Seifer and Mataric’s (2005) definitions of SARs as “the intersection of Assistive Robots and Socially Interactive Robots”. To the best of our knowledge, this is the first SAR platform specifically developed for use by IwPD.

The results of Phase 2 of the feasibility study, compared to Phase 1, suggest an increase in challenge (as hinted to by the trend for slower reaction times in two out of the three exercise sets (Figure 6), and increased time spent at lower difficulty levels in both the KB and TL exercise sets (Figure 8)) without affecting the median enjoyment level (Figure 9). This suggests that the most updated prototype provided a suitable level of challenge (not too easy on the one hand, and not frustratingly challenging on the other) for the participants and maintained a balance between difficulty and engagement.

Motor, cognitive, musical and social elements were brought up by clinicians in all parts of this experiment, requested by IwPD in Phase 1, and implemented in the system upgrade between Phases 1 and 2. These factors likely contributed to the higher long-term acceptance of the system in Phase 2 compared to Phase 1 (80% vs. 67%), the lower level of reported frustration in Phase 2 compared to Phase 1 (Figure 9), as well as to the statements made by two participants, that their symptoms were alleviated after exercising with the platform.

The experimenters anecdotally noted that some of the participants danced, sang along with the music or whistled during training. They also noted the participants responded to the auditory feedback given by the robots - often cheering when the robot cheered, or talking back to the robot in case they received negative feedback. Participants who were able to safely do so stood up during the training session, which appeared to indicate a high engagement level.

Based on the in-depth interviews conducted in Section 3.3.2, two adaptations can be considered for future versions of the system. First, creating an interface that lets participants easily activate the system by themselves. This can increase the individual’s sense of capability, which for many IwPD is significantly decreased when facing the many struggles that appear with the disease (Prenger et al., 2020). Second, to emphasize the sociability factor of the robot so people can use it with other human partners while practicing.

Other suggestions that were not implemented in the current version of the system are a learning algorithm that learns the songs the patient likes, in order to maintain novelty and interest, adding a sing-along mode for a higher level of difficulty which also improves breathing and vocal cord strength and a mode requesting a specific clicking hand for crossover training and design the setup on a telescopic table to train the legs and work on balance.

### 4.1 Design insights for technologies for IwPD

The feedback we collected in this study on a system for IwPD has shed light on design aspects, which we anticipate may be helpful beyond this specific platform, for other technologies designed for IwPD, and potentially also for technology design for healthcare more broadly. We identified the following aspects: 1) the *size* of the platform appeared to be a crucial factor in the willingness to use the technology in the long term in the home setting; this was brought up by half of the participants; 2) the *simplicity* of the interface (Feingold Polak and Weiss, 2023) and of the practice explanations was important to enable most IwPD to understand the tasks and to be able to participate effectively; 3) the *novelty effect* is an important issue to consider with a platform designed for long-term use: this is a topic that the participants in this study brought up themselves, and is a crucial aspect of successful adoption of the technology; 4) the *social aspect of the interaction* with the robot seemed to be an important motivational factor, as evidenced by the repeated requests by participants to add competitive exercise modes with other participants, additional robot speech capabilities, and a more animated physical design; 5) the *participatory design* approach, in which the intended end users of the system–as well as members of their support network–provide feedback on it, to inform the iterative design process, did not only mean that we were able to design a platform that better suited their needs, but also helped the participant recruitment effort, since our participants indicated they were highly motivated to contribute to the improvement of technological solutions for their community. Indeed, when reflecting on the entire process, we were struck by the difference between our initial suggested design and the actual prototype 3.0, which stresses the effectiveness of the participatory design process.

### 4.2 Study limitations

In the feasibility study, our participants interacted with the platform during a single session, which restricted our ability to assess the potential long-term improvements in cognitive and motor abilities when using the platform. Furthermore, conducting the experiment under the conditions of a controlled laboratory setting, rather than in the participants’ home environment or clinical settings, might have impacted participants’ perception of the system.

## 5 Conclusion

We developed and tested a SAR platform for cognitive-motor exercise by individuals with IwPD. The system incorporated gamified exercises to train cognitive and motor abilities. Feasibility testing with 16 IwPD showed positive engagement and motivation to use the platform in the short term. The platform is novel and unique in its targeted approach of using SARs for IwPD and incorporates interactive gamified elements. In the long run, it aims to improve cognitive and motor functionality in IwPD to potentially help manage some of the PD symptoms. The study assessed technical requirements, user acceptance, operational challenges, and safety considerations. Participants reported high enjoyment levels, and willingness to continue training. Future directions include long-term studies to assess the system’s impact on cognitive and motor abilities over time. The platform is designed to complement and support the work of clinicians by providing engaging and motivating training for IwPD.

## Data Availability

The original contributions presented in the study are included in the article/Supplementary Material, further inquiries can be directed to the corresponding author.
