# Supplementary material for: A novel socially assistive robotic platform for cognitive-motor exercises for individuals with Parkinson's Disease: a participatory-design study from conception to feasibility testing with end users
Source: Front Robot AI. 2023 Oct 6;10:1267458. doi: 10.3389/frobt.2023.1267458 (PMC10587405; doi:10.3389/frobt.2023.1267458)
Supplement: Supplementary file 2 [file Table1.DOCX]

# Hardware

The robot-based exercise platform uses two mobile robots and a computer. Each robot (sized 168x165x268 mm) has a TurtleBot3 Burger mobile robot core, equipped with an LED-matrix (SparkFun LuMini LED 8x8 Matrix) for visual cues to users, a tracking camera (Intel® RealSense™ Tracking Camera T265) for localization in space (see Figure S1). It is also equipped with a custom-made semi-transparent casing optimized for size, shape, and transparency, designed to endow the robot with a pleasant look, while enabling the color cues of the LEDs to show through the casing (see Figure 2).


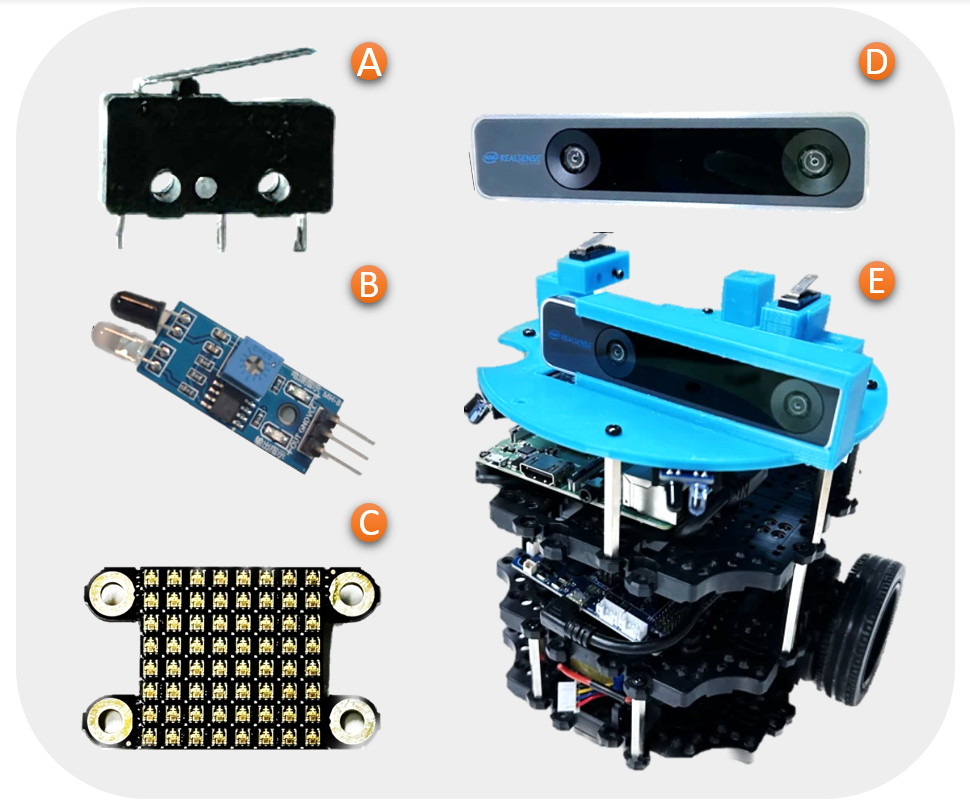


Figure S1. The sensors used on the robotic platform.

(A) A CNLW 3P microswitch (B) A Waveshare Infrared Proximity Sensor (C) An 8x8 Sparkfun LuMini LED matrix (D) An Intel Realsense T265 tracking camera. (E) The Turtlebot3 equipped with the sensors

The main components of the robotic platform include:

## **Turtlebot3**

Turtlebot3 (Tully Foote and Melonee Wise, Willow Garage) is a small (138mm x 178mm x 192mm), programmable mobile robot, which uses the Robot Operating System (ROS). It contains a Raspberry Pi 3b+ Single Board Computer (SBC), suitable for robust embedded applications.

## **Intel® Realsense™ Tracking Camera T265**

This device uses V‐SLAM technology, which combines cameras and inertial measurement units (IMU) to navigate and keep track of its location. It is equipped with two fisheye lens sensors, an IMU and an Intel® Movidius™ Myriad™ 2 Vision Processing Unit (VPU).

## **SparkFun LuMini LED Matrix**

A SparkFun LuMini LED Matrix has 64 individually addressable LEDs that can each produce 16 million colors.

## **Waveshare Infrared Proximity Sensor**

A 39mm×15.5mm sensor which emits a beam of infrared light and outputs a collision based on the reflected beam, contingent on a custom-set threshold.

The components are connected to a Raspberry Pi board as laid out in Figure S2.


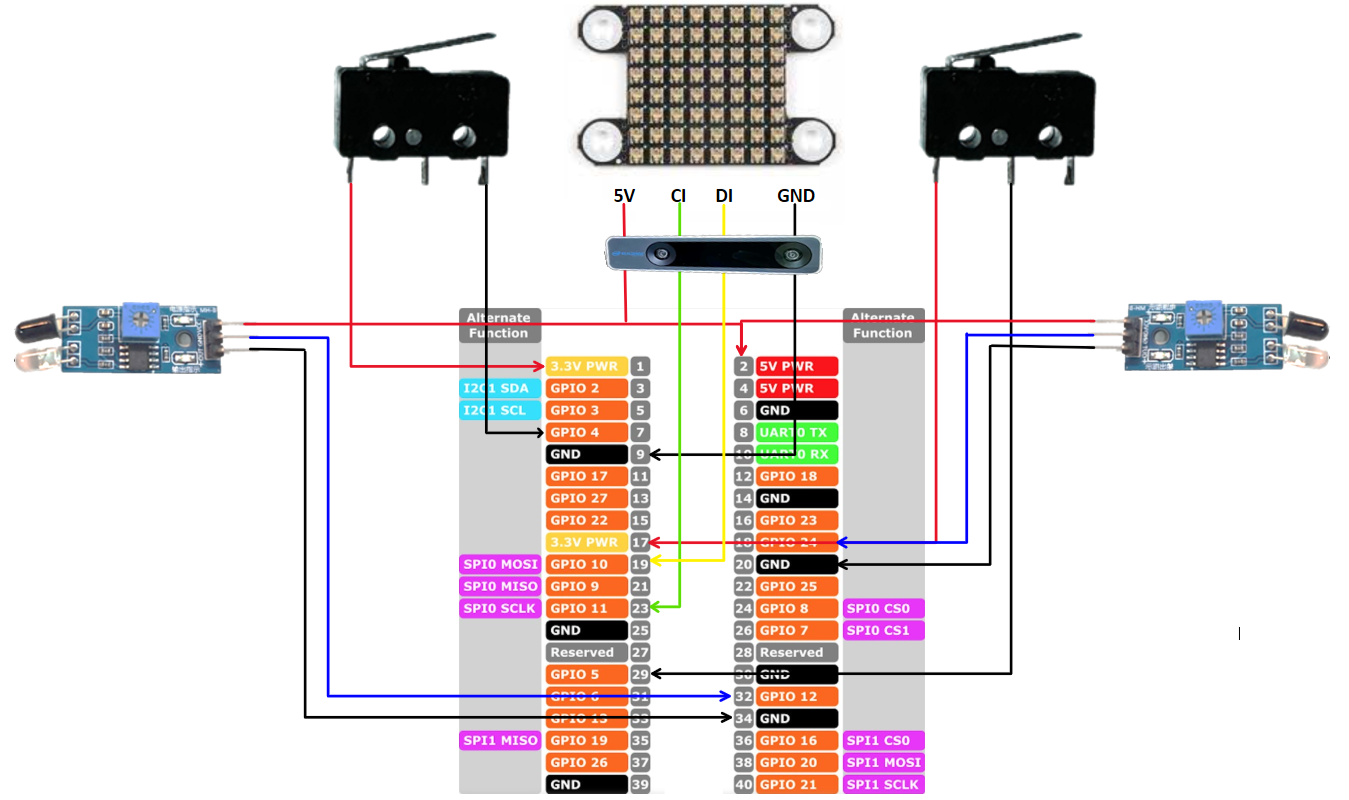


Figure S2. Connection scheme between the sensors and the Raspberry Pi

# Software

This section describes the different robot software modules developed for the platform, as well as the high-level game logic that orchestrates the actions associated with each exercise.

# Robot modules

The robot software integrates the different hardware components and ensures their synchronous operation through the Robot Operating System (ROS) software libraries, using custom-written code. This software module consists of several key functionalities, including translation of robot movement commands (e.g., "move straight") into smooth motor commands, as well as accurate location measurement and trajectory tracking using IMUs, wheel and visual odometry. It also allows precise control of the LED matrix with predefined colors and dynamic blinking patterns, detection of presses on the robot head, handling collisions using two proximity sensors, and robust noise filtering for sensor data. In addition, the modules include a status indicator that indicates whether a robot is online during an exercise, as well as an automated reconnection method.

# Results

Table S1: Suggestions made by clinicians during the iterative design process grouped into themes

|  | Part 1  **In-depth interviews with clinicians** | Part 2  **Prototype 1.0 and follow-ups with a clinician** | Part 3  **Brainstorming session with clinicians** |
| --- | --- | --- | --- |
| **User interface and experience** | (1) Simplify and minimize the information displayed on the screens and only provide stimuli that are necessary for the game. Otherwise, they will be distractors.  (2) Avoid scores out of 100. Prefer time-dependent scores, and ones that do not induce self-judgment and self-criticism. | (1) Make the background of the screens of the computer screen less distracting.  (2) Increase font size on the computer screen. | Remove the distracting background music in the Simon Says exercise |
| **Customization and flexibility** | (1) Enable user-specific adaptations to the exercise parameters (number of clicks, time to click, time between clicks, colorfulness), so the user gets just the right challenge. The right challenge will prevent frustration from the exercise being too easy or too distracting and difficult.  (2) Allow multiple song choices at different speeds for added variability. | N/A | (1) Make the Traffic Light exercise more challenging; In response, we increased the rate at which the lights are turned on and off and the variability of the delay between light-ups.  (2) Add more song choice for the users |
| **Design and aesthetics** | Make the robots non-anthropomorphic and without human-like facial gestures which may be perceived as scary | N/A | N/A |
| **Music and audio feedback** | N/A | (1) Add a 'good job' / 'oops' sound for immediate audio feedback.  (2) Make the songs in Hebrew and slower to better fit them to the population.  (3) Prefer on-beat music with pronounced beats, as in march music. | (1) Add host-like responses from the robot which greet the user and invite them to use the system  (2) Change the physical setup to make the music come from the front of the user instead of the side |
| **Exercise modes** | Slow down the exercise pace. PD patients have a slower reaction time relative to healthy individuals. | Implement a Simon Says mode which focuses on executive function session. | (1) Improve the synchronization between the robots' movements and the music and vary the movement patterns.  (2) Simplify the Simon Says tutorial and further clarify the rules |
| **data visualization** | N/A | Create a graph of the reaction times throughout the session for the clinicians. | N/A |
| **experimental design** | N/A | Adjust the inclusion and exclusion criteria for the experiment criteria (Hoehn and Yahr 2 and below, use the MoCA, recruit only people with Idiopathic ' 'Parkinson's Disease and without symptoms of ' 'Parkinson's Plus etc.) | N/A |
| **safety** | N/A | (1) Ensure the experimental setup area does not pose a risk of falling.  (2) Design the workstation so that the pressing angle maximizes the physiological benefits of practice while minimizing strain on the elbows. | N/A |
| **Ideas which we did not implement** | (1) a learning algorithm that learns the songs the patient likes, to maintain novelty and interest  (2) add a sing-along mode for a higher level of difficulty which also improves breathing and vocal cord strength  (3) ask the participant to use a specific hand when pressing down on the robot, for crossover training, and design the setup on a height-adjustable table to train the legs and work on balance. | N/A (all suggested changes were implemented in the system) | (1) The users should exercise with alternating hands on a height-adjustable table  (2) The robots should be placed on the ground, the user should follow them, and step on them in order to train their balance and improve their leg strength  (3) The robots should climb up and down walls with the user standing in front of them rather than move laterally on a table with the user sitting down, to work on balance  (4) The users should use their voices to make the robots move; This suggestion was made by a speech and language therapist, which further suggested that the exercises should be collaborative, with two users working together, and the recognition of each ' 'user's voice would lead to different functionality of the robots (e.g., moving forward based on one ' 'user's voice, and backwards based on another's)  (5) The robots should take on different embodiments, (e.g., a cat and mouse). |

Video S1. IwPD performing the ‘King of the Bongos’ exercise set with the robotic platform we developed

Video S2. IwPD performing the ‘Traffic Light’ exercise set with the robotic platform we developed

Video S3. IwPD performing the ‘Simon Says’ exercise set with the robotic platform we developed
